# BCG-Induced Trained Immunity in Healthy Individuals: The Effect of Plasma Muramyl Dipeptide Concentrations

**DOI:** 10.1155/2020/5812743

**Published:** 2020-06-15

**Authors:** Vera P. Mourits, Valerie A. C. M. Koeken, L. Charlotte J. de Bree, Simone J. C. F. M. Moorlag, Wern Cui Chu, Xiaoli Xu, Helga Dijkstra, Heidi Lemmers, Leo A. B. Joosten, Yue Wang, Reinout van Crevel, Mihai G. Netea

**Affiliations:** ^1^Department of Internal Medicine, Radboud Institute of Molecular Life Sciences (RIMLS) and Radboud Center for Infectious Diseases (RCI), Radboud University Medical Center, 6525 Nijmegen, Netherlands; ^2^Research Center for Vitamins and Vaccines, Bandim Health Project, Statens Serum Institut, 2300 Copenhagen, Denmark; ^3^Odense Patient Data Explorative Network, University of Southern Denmark/Odense University Hospital, 5000 Odense, Denmark; ^4^Institute of Molecular and Cell Biology, Agency for Science, Technology and Research, Queenstown, Singapore 138673; ^5^Department of Medical Genetics, Iuliu Hatieganu University of Medicine and Pharmacy, 400012 Cluj-Napoca, Romania; ^6^Department of Biochemistry, Yong Loo Lin School of Medicine, National University of Singapore, Queenstown, Singapore 119228; ^7^Department for Genomics & Immunoregulation, Life and Medical Sciences Institute (LIMES), University of Bonn, 53115 Bonn, Germany

## Abstract

BCG vaccination protects not only against tuberculosis but also against heterologous infections. This effect differs between individuals, yet the factors responsible for this variation are unknown. BCG-induced nonspecific protection is, at least partially, mediated by innate immune reprogramming (trained immunity), which can be induced by the muramyl dipeptide (MDP) component of peptidoglycans. We aimed to study whether differential release of MDP in healthy individuals may explain variability of their response to BCG vaccination. Circulating MDP concentrations were increased up to three months after BCG vaccination. MDP concentrations at baseline, but not their increase postvaccination, positively correlated with the induction of trained immunity and not with mycobacteria-induced T-cell responses. Interestingly, MDP concentrations correlated with inflammatory markers in the circulation. In conclusion, circulating MDP concentrations are associated with the strength of trained immunity responses and thus influence the biological effects of BCG vaccination. This study increases our understanding about the role of MDP in BCG-induced trained immunity, which might help to optimize vaccine efficacy and explore novel applications of BCG vaccination.

## 1. Introduction

The Bacillus Calmette-Guérin (BCG) vaccine is a live-attenuated vaccine that protects against tuberculosis, one of the world's deadliest infectious diseases [1]. In the last decades, it has been shown that BCG vaccination, besides its effects for the prevention of tuberculosis, reduces all-cause morbidity and mortality in children and neonates, especially by protecting against heterologous infections [2]. This nonspecific protection could, at least partially, be explained by induction of trained immunity [3]. This is a process of innate immune cell reprogramming, a de facto nonspecific innate immune memory, in which certain infections and vaccinations induce an enhanced inflammatory response in innate immune cells upon restimulation with an unrelated infectious agent.

BCG vaccination induces trained immunity in monocytes and natural killer cells, which leads to increased expression of activation markers and production of proinflammatory cytokines in response to *Staphylococcus aureus* or *Candida albicans* [4–6]. The induction of trained immunity in monocytes is mediated through extensive metabolic and epigenetic reprogramming and persists even one year after BCG vaccination in humans [4, 5, 7]. Recently, it has been shown that BCG-induced trained immunity acts via modulation of hematopoietic stem and progenitor cells (HSPCs), explaining the persistence of these effects [8, 9]. Trained immunity responses induced by BCG vaccination were shown to be dependent on the engagement of the intracellular receptor nucleotide-binding oligomerization domain 2 (NOD2) by the peptidoglycan component muramyl dipeptide (MDP) [5].

MDP was discovered as the minimal bioactive component required for Freund's Complete Adjuvant [10]. MDP is present in human peripheral blood [11] and is derived from different sources. Bacteria or bacterial components could directly affect circulating MDP concentrations, but also bacteria in the microbiome produce peptidoglycans (PGNs) which can be translocated from the intestinal mucosa into the circulation [11, 12]. MDP interacts with NOD2 in the cytoplasm of immune cells [13] and thereby induces a signaling pathway resulting in the production of chemokines and cytokines [14]. Interestingly, innate immunity to mycobacterial infection in mice is significantly reduced in NOD2-deficient cells [15]. Furthermore, it has been shown that *N-*glycolylated MDP contributes to the immunogenicity of *Mycobacterium tuberculosis* [16].

**Figure 1:**
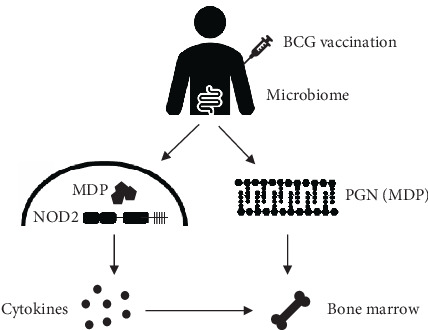
The proposed role of MDP in trained immunity. Upon BCG vaccination, MDP binds to NOD2 in innate immune cells, thereby inducing cytokine production in the periphery, which could indirectly induce long-term reprogramming of bone marrow hematopoietic stem and progenitor cells (HSPCs). Alternatively, MDP may directly access the bone marrow to skew HSPCs, which is also influenced by MDP released by the microbiome.

In this study, we aimed to investigate whether variation in circulating MDP concentrations modulates trained immunity responses induced by BCG vaccination in vivo and may explain the variability of the response between individuals. Two possible mechanisms for a role of MDP in BCG vaccination-induced trained immunity are proposed (Figure 1): BCG vaccination could induce the production of cytokines in the periphery, which could indirectly induce long-term reprogramming of bone marrow HSPCs, or MDP may directly access the bone marrow and prime HSPCs.

## 2. Methods

### 2.1. Study Cohort

To study the immunological effects of MDP on BCG vaccination, 325 healthy volunteers of Western European ancestry included in the 300BCG study between April 2017 and June 2018 received a standard dose of 0.1 mL BCG (Bulgaria strain, InterVax, Canada) intradermally. Afterwards, four individuals were excluded from the study due to medication use. Blood was collected before and two weeks and three months after BCG vaccination. Volunteers were excluded upon the following criteria: use of systemic medication other than oral contraceptives and acetaminophen, use of antibiotics three months before inclusion, previous BCG vaccination, history of tuberculosis, any febrile illness four weeks before participation, vaccination three months before participation, and a medical history of immunodeficiency. Health was monitored in short interviews during their visits. Eighteen participants were excluded before performing analyses comparing before and after BCG vaccination since they were vaccinated at a different time during the day, as part of a separate substudy. The study was approved by the Arnhem-Nijmegen medical ethical committee (NL58553.091.16) and performed in accordance with the Declaration of Helsinki. All volunteers had given written informed consent before they underwent any research procedure.

### 2.2. MDP Measurements

Indirect competitive enzyme-linked immunosorbent assay (icELISA) was used for muramyl-l-alanyl-d-isoglutamine (MDP) quantification, as described previously [11]. In short, coating solution (1 *μ*g/mL MDP-human serum albumin in coating buffer) was added to ½-area 96-well plates (Costar, Corning) and incubated at 4°C overnight. Thereafter, blocking buffer was added for 1 h at 37°C, plate was washed, and a mixture of 2E7 solution and the samples (ratio 3 : 1) were added to the plate and incubated for 2 h. After washing, a secondary antibody (HRP-conjugated anti-mouse IgG antibody (GE Health, 1 : 2000)) was added and incubated for 1 h, followed by washing. Then, the 1-Step™ Ultra TMB-ELISA substrate solution (Thermo Fisher Scientific) was added for 5 minutes, and 2 M sulfuric acid solution was thereafter added to terminate the reaction. Absorbance was read using a microtiter plate reader (Tecan Infinite Pro2000). Samples were measured in duplicate, and no samples were below the detection limit (100 ng/mL).

### 2.3. PBMC Isolation and Stimulation

Peripheral blood mononuclear cells (PBMCs) were isolated from EDTA whole blood by density centrifugation on Ficoll-Paque (GE Healthcare), washed three times in PBS and resuspended in RPMI 1640 medium (Dutch modified) (Gibco, Life Technologies), supplemented with 5 *μ*g/mL gentamycin, 2 mM Glutamax (Gibco), and 1 mM pyruvate (Gibco), and supplemented with 10% human pooled serum for cultures stimulated for 7 days. After isolation, 5 × 10^5^ PBMCs were added to a round bottom 96-well plate (Greiner) and stimulated with RPMI (control), 5 *μ*g/mL *M. tuberculosis* HR37v, or heat-killed *S. aureus* (10^6^/mL). Cells were incubated for 24 hours or 7 days at 37°C, 5% CO_2_, and thereafter, supernatant was collected and stored at -20°C until further analysis. Complete blood counts were performed on EDTA whole blood and PBMC fractions after Ficoll isolation, on the Sysmex XN-450 hematology analyzer.

### 2.4. Cytokine Measurements

IL-1*β*, IL-6, and TNF-*α* production was determined in 24-hour supernatants by using ELISA (R&D Systems and Sanquin, Amsterdam). In proof-of-principle experiments performed in a subgroup of volunteers, we measured TNF-*α* upon ex vivo *M. tuberculosis* stimulation. Because the vast majority of TNF-*α* concentrations were below detection limit, TNF-*α* production after *M. tuberculosis* stimulation was not assessed in all samples. IFN-*γ* production after 7 days and IL-1*β* production upon *M. tuberculosis* stimulation were determined by using Luminex (ProcartaPlex Thermo Fisher), according to the manufacturers' protocol.

### 2.5. Measurements of Circulating Inflammatory Mediators

The commercially available Proseek Multiplex Inflammation I panel (Olink Proteomics, Uppsala, Sweden) was used to measure a panel of 92 inflammation-related proteins in EDTA plasma. The procedure of the multiplex proximity extension assay was performed as previously described [17]. Briefly, proteins are recognized by pairs of antibodies coupled to cDNA strands. These cDNA strands bind when they are in close proximity, after which they extend and amplify by a polymerase reaction. After detection and normalization, this results in normalized protein expression values, measured on a log_2_ scale. Proteins were excluded from the analysis when the target protein was detected in less than 75% of the samples.

### 2.6. Statistical Analyses

Data was analyzed using Wilcoxon signed-rank test for paired samples, a Mann–Whitney U test for unpaired samples, or Spearman correlation using GraphPad Prism software (GraphPad, version 5.03) and R (3.3.3). No correction for multiple testing was applied as this is an exploratory research with expected small effect sizes, and only one factor (MDP concentration) was assessed, with a clear biological hypothesis. Data are expressed as mean ± SEM, and values of ∗*P* < 0.05, ∗∗*P* < 0.01, and ∗∗∗*P* < 0.001 were considered statistically significant.

## 3. Results

### 3.1. BCG Increases Circulating MDP Concentrations

**Figure 2:**
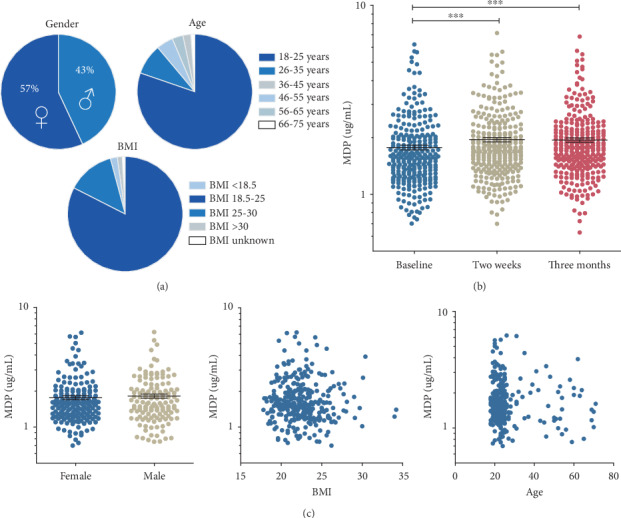
Circulating MDP concentrations are elevated in BCG-vaccinated individuals. (a) Participant characteristics of the study cohort (*n* = 321) (BMI = body mass index). (b) MDP concentrations in plasma of participants of which complete data was available, before, two weeks, and three months after BCG vaccination, using icELISA (mean ± SEM, *n* = 288, ∗∗∗*P* < 0.001, Wilcoxon signed-rank test). (c) The concentrations of MDP at baseline did not correlate to sex (mean ± SEM, *n* = 316, Mann–Whitney U test), age, or BMI (*n* = 316, Spearman correlation).

MDP concentrations in the circulation were measured in the serum of 321 healthy individuals of the 300BCG cohort (aged 18-75 years, 57% women, 83% body mass index (BMI) between 18.5 and 25 (Figure 2(a))). MDP concentrations in the individuals were measured in serum just before BCG vaccination (baseline) and two weeks and three months after vaccination. As seen in Figure 2(b), MDP concentrations in the circulation were significantly increased after BCG vaccination, both two weeks and three months postvaccination. MDP concentrations at baseline were not affected by age, BMI, or sex (Figure 2(c)). MDP concentrations two weeks or three months after vaccination were also not affected by these factors (data not shown).

### 3.2. Circulating MDP Correlates with Red Blood Cell Counts and Systemic Inflammation

**Figure 3:**
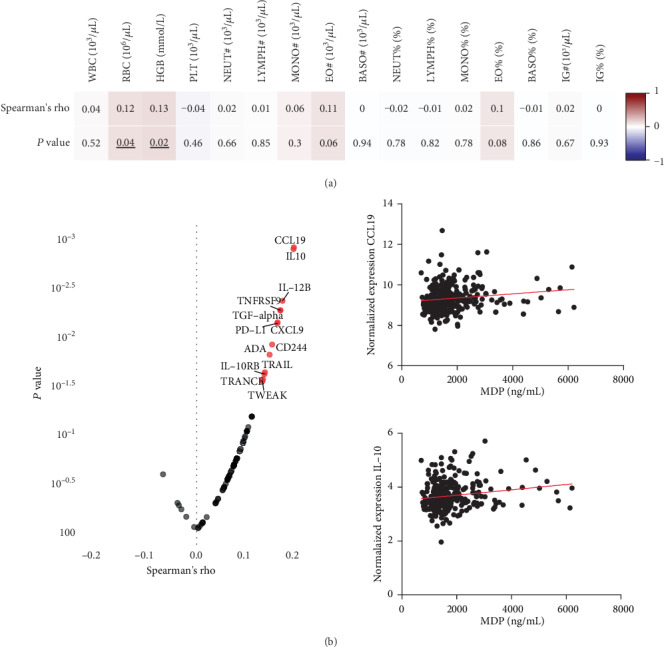
The concentration of circulating MDP is positively correlated with the concentration of red blood cells and systemic inflammation in peripheral blood. (a) Spearman correlation of circulating MDP concentrations at baseline and various cell subsets measured in whole blood on Sysmex hematology analyzer (*n* = 316). V1 = visit 1 (before vaccination); V2 = visit 2 (two weeks after vaccination); V3 = visit 3 (three months after vaccination). WBC = white blood cell; RBC = red blood cells; HGB = hemoglobulin; PLT = platelets; # = count; NEUT = neutrophil; LYMPH = lymphocytes; MONO = monocytes; EO = eosinophils; IG = immunoglobulin. (b) Spearman correlation of circulating MDP concentrations and immunomodulators at baseline in peripheral blood by using Olink platform (*n* = 313). Two examples of correlations with MDP are shown (CCL19 and IL-10). Cells of both rho and *P* value are colored based on rho value. Significant changes are underlined.

To study whether MDP is associated with specific cell populations, MDP concentrations were correlated with different cell subsets in the circulation. As seen in Figure 3(a), MDP concentrations are positively correlated with the erythrocyte numbers and hemoglobin levels. Next, MDP concentrations were correlated with immune modulators such as chemokines and cytokines in peripheral blood. Of the 73 markers of inflammation we investigated in our analyses, 13 were significantly associated with MDP concentrations. As seen in Figure 3(b), MDP concentrations are positively correlated with normalized expression values of natural killer cell receptor 2B4 (CD244), TNF-related apoptosis-inducing ligand (TRAIL), CXCL9 (or MIG), TGF-alpha, CCL19, IL-10 receptor subunit beta (IL10-RB), programmed cell death 1 ligand 1 (PD-L1), TNF-related activation-induced cytokine (TRANCE/TNFSF11), IL-12 subunit beta (IL-12B), IL-10, TNF receptor superfamily member 9 (TNFRSF9), TNFRSF12 (TWEAK), and adenosine deaminase (ADA), of which two examples are shown. See Figure S1 in the Supplementary Materials for individual correlation plots. This suggests that the amount of MDP in the circulation is associated with the inflammatory status of an individual.

### 3.3. Baseline MDP Concentrations Are Associated with BCG-Induced Trained Immunity Responses

**Figure 4:**
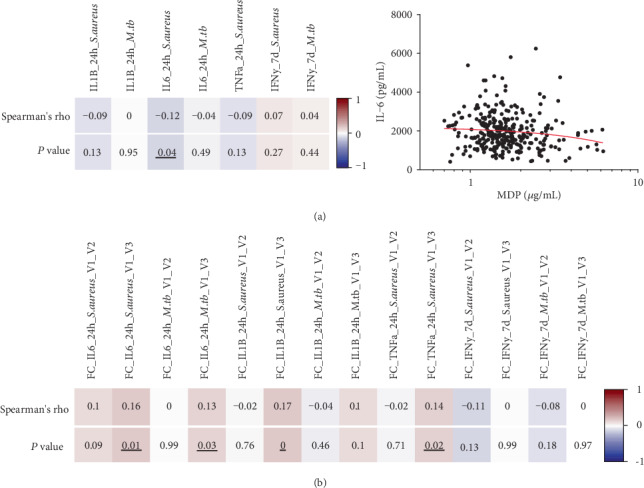
The strength of trained immunity responses upon BCG vaccination is associated with circulating MDP concentrations at baseline. (a) Spearman correlation of circulating MDP concentrations before BCG vaccination and fold changes of cytokine production before versus after vaccination upon ex vivo stimulation with *S. aureus* and *M. tuberculosis (M. tb)* (*n* ≥ 251). Significant correlation between IL-6 production and MDP concentrations is shown. (b) Spearman correlation of circulating MDP concentrations at baseline and fold changes of cytokine production to *S. aureus* and *M. tuberculosis* restimulation after BCG vaccination (*n* ≥ 205, Spearman correlation). V1 = visit 1 (before vaccination); V2 = visit 2 (two weeks after vaccination); V3 = visit 3 (three months after vaccination). Cells of both rho and *P* value are colored based on rho value. Significant changes are underlined.

Since MDP is able to induce chemokine and cytokine production in vitro, also in synergy with other stimuli [14], we investigated whether MDP concentrations in vivo correlate with cytokine production upon ex vivo PBMC stimulation with the nonspecific stimulus *S. aureus* or specific stimulus *M. tuberculosis*. MDP concentrations in vivo were moderately negatively associated with the production of proinflammatory cytokine IL-6 upon 24 hours of stimulation with *S. aureus* (Figure 4(a)), but association was not observed with other stimuli and cytokines. On the other hand, MDP baseline concentrations more strongly predicted trained immunity responses. Circulating MDP concentrations in the individuals at baseline were correlated with changes in cytokine responses before (V1) versus two weeks (V2) and three months (V3) after BCG vaccination upon ex vivo restimulation with *S. aureus* and *M. tuberculosis*, representing, respectively, trained immunity responses and specific responses. Whereas no significant correlation between MDP concentrations and the immune responses to *M. tuberculosis* responses was observed, MDP concentrations at baseline were positively correlated with the heterologous *S. aureus*-induced IL-1*β*, IL-6, and TNF-*α* production three months after BCG vaccination (Figure 4(b)). See Figure S2 in the Supplementary Materials for individual correlation plots. This indicates augmented trained immunity responses in individuals with higher basal MDP concentrations in the circulation. MDP concentrations do not correlate with scar size two weeks or three months after BCG vaccination (data not shown).

### 3.4. Changes in MDP Concentrations after BCG Vaccination Do Not Correlate with Trained Immunity Responses

**Figure 5:**
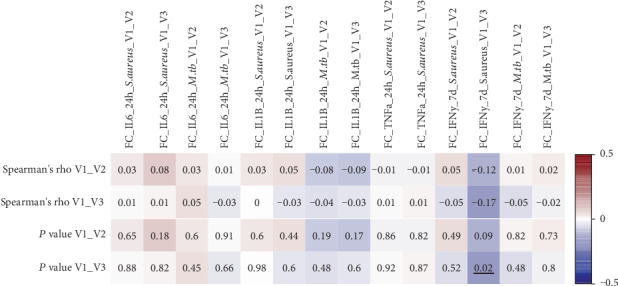
Changes in MDP circulating concentrations after BCG vaccination do not correlate with trained immunity responses. Spearman correlation of MDP fold changes before versus after BCG vaccination correlated with fold changes of cytokine production before versus after BCG vaccination upon ex vivo stimulation with *S. aureus* and *M. tuberculosis* (*n* ≥ 205, Spearman correlation). V1 = visit 1 (before vaccination); V2 = visit 2 (two weeks after vaccination); V3 = visit 3 (three months after vaccination). Cells of both rho and *P* value are colored based on rho value. Significant changes are underlined.

To study whether the change in MDP concentration following BCG vaccination also influences trained immunity, the ratio between MDP concentrations before and after BCG vaccination was correlated with the ratio of the cytokine responses before and after BCG vaccination (Figure 5). No correlations were observed between the fold changes of MDP and cytokines after BCG vaccination, suggesting that the increase in circulating MDP upon BCG vaccination does not impact trained immunity responses. However, a significant negative correlation was observed between increase in MDP and IFN-*γ* fold changes upon *S. aureus* restimulation (Supplementary Figure 3B).

## 4. Discussion

The heterologous protective effects of BCG are important in countries with a high infectious burden, yet these effects are variable across different settings and populations. The factors influencing this variability are unknown. In this study, we show that circulating concentrations of MDP, the active component of mycobacterial peptidoglycans, are positively correlated with induction of trained immunity and therefore is likely to affect the heterogeneity of these innate immune responses.

MDP can be found in the circulation of healthy volunteers [11], but the role of MDP in modulating immune responses in vivo is incompletely understood. Interestingly, we show that circulating MDP concentrations in healthy individuals are positively correlated with red blood cell counts, as well as various inflammatory biomarkers in peripheral blood. This important finding implies that steady-state MDP, probably originating from the gut microbiome, may regulate systemic inflammation. This means that peptidoglycans, next to lipopolysaccharide (LPS), should be considered an important exogenous mediator to be followed in various clinical conditions associated with impaired intestinal barrier or chronic low-grade inflammation. It is not fully known which are the mechanisms through which MDP enters the cell and activates NOD2, but several suggestions include internalization of MDP by phagocytosis of whole bacteria, endocytosis, and uptake from outer membrane vesicles, transmembrane channels, or peptide transporters [18].

Steady-state circulating concentrations of MDP contributed to the explanation of interindividual variation in trained immunity responses induced by BCG vaccination, while it only mildly affected cytokine production capacity prior to BCG vaccination. The exact mechanism through which MDP concentrations could influence trained immunity remains to be elucidated, although epigenetic rewiring through NOD2 engagement [5] is likely to play an important role. Interestingly, the specific immune response (*M. tuberculosis*-induced IFN-*γ* production) was not influenced by MDP concentrations, indicating different mechanisms regulating specific immune memory and trained immunity.

BCG vaccination increases MDP concentrations in the circulation up to at least three months after vaccination, probably through release of peptidoglycans from the local site of vaccination. It is unlikely that this effect is due to spurious variation, since a previous study showed that circulating MDP levels are relatively stable over time [11]. Interestingly, however, this (moderate) additional increase did not further modulate trained immunity: it is thus likely that the epigenetic changes underlying trained immunity are relatively early events, as demonstrated earlier [5], and that later changes in circulating peptidoglycans under the influence of local release from the site of vaccination have little additional impact.

Peptidoglycan MDP has multiple roles in the modulation of immune responses. van der Meer et al. showed that MDP affects cytokine production in isolated human mononuclear cells, but not in a whole-blood assay in vitro. Since this lack of effect was independent of plasma-derived factors, it has been suggested that a cellular or cell-derived component from the blood is responsible for the inhibition of MDP in whole blood [19]. Furthermore, although MDP alone cannot induce immunoglobulin production [20], it is able to enhance expression of cell adhesion molecules and cytokines [14]. Moreover, several studies show increased production of proinflammatory cytokines upon MDP stimulation in vitro, also in synergy with other stimuli, which seem to depend on species and cell types used [14, 19, 21, 22]. It is known that oxidatively modified erythrocytes and erythrocyte-derived free hemoglobin augment cytokine release from human monocytes by bacterial stimuli such as LPS [23–25]. Here, we observe a positive correlation between MDP and red blood cell concentrations, as well as hemoglobin levels. In contrast, free hemoglobin in vitro has been shown to inhibit the effect of MDP upon LPS-induced cytokine production [19]. Besides that, it has been shown that the addition of neutrophils together with MDP in vitro decreases LPS-induced cytokine production [14], whereas we do not observe an association between neutrophils, or other cell subsets, and MDP. To this list of effects, we now report that steady-state MDP plasma concentrations have an important impact on trained immunity responses induced by BCG.

As previously mentioned, the microbiome is an important source of circulating MDP [26]. Huang et al. showed that PGN is barely detectable in serum of germfree mice, in contrast to specific-pathogen-free mice, suggesting that a large proportion of PGN in the circulation is derived from the microbiome [11]. Additionally, Clarke et al. showed that PGN from the microbiota by NOD1 enhances systemic innate immunity in mice [12]. Therefore, in the future, it would be interesting to observe the influence of the human microbiome on circulating MDP and trained immunity responses as well.

## 5. Conclusions

In conclusion, circulating MDP concentrations prior to vaccination correlate with systemic inflammation and induction of trained immunity following BCG vaccination, but not with specific T-cell cytokine responses. Additionally, BCG vaccination leads to a sustained increase in circulating MDP concentrations, but this change in MDP does not affect trained immunity responses or specific memory immune responses. This study increases our understanding about the role of MDP in BCG-induced trained immunity, which might help to optimize vaccine efficacy and explore novel applications of BCG vaccination.

## Data Availability

The data used to support the findings of this study, which are not included in the article, are available from the corresponding author upon request.

## Supplementary Materials

Supplementary Materials Supplementary Figure 1:Circulating MDP is positively correlated with the concentration of red blood cells and systemic inflammation. (A) Hemoglobin (HGB, mmol/L) and (B) amount of red blood cells (RBC, 10^6/*μ*L) measured on Sysmex hematology analyzer correlated with MDP concentrations at baseline (*n* = 316). (C) Spearman correlation of circulating MDP concentrations and immunomodulators at baseline in peripheral blood by using Olink platform (*n* = 313). Supplementary Figure 2: Baseline MDP concentrations are associated with BCG-induced trained immunity responses. Spearman correlation of circulating MDP concentrations at baseline and fold changes of IL-1*β*, IL-6, and TNF-*α* production to *S. aureus* stimulation three months after BCG vaccination (*n* ≥ 289). Supplementary Figure 3: Changes in MDP concentration upon BCG vaccination correlate with changes in IFN-*γ* production upon ex vivo *S. aureus* stimulation. Circulating MDP concentrations before versus three months after BCG vaccination are correlated to IFN-*γ* production after 7 days upon ex vivo *S. aureus* stimulation (Spearman correlation, *n* = 212).

## References

[1] (2018). *Global tuberculosis report*.

[2] Butkeviciute E., Jones C. E., Smith S. G. (2018). Heterologous effects of infant BCG vaccination: potential mechanisms of immunity. *Future Microbiology*.

[3] Netea M. G., Joosten L. A. B., Latz E. (2016). Trained immunity: a program of innate immune memory in health and disease. *Science*.

[4] Kleinnijenhuis J., Quintin J., Preijers F. (2014). Long-lasting effects of BCG vaccination on both heterologous Th1/Th17 responses and innate trained immunity. *Journal of Innate Immunity*.

[5] Kleinnijenhuis J., Quintin J., Preijers F. (2012). Bacille Calmette-Guerin induces NOD2-dependent nonspecific protection from reinfection via epigenetic reprogramming of monocytes. *Proceedings of the National Academy of Sciences of the United States of America*.

[6] Kleinnijenhuis J., Quintin J., Preijers F. (2014). BCG-induced trained immunity in NK cells: role for non-specific protection to infection. *Clinical Immunology*.

[7] Arts R. J. W., Carvalho A., La Rocca C. (2016). Immunometabolic pathways in BCG-induced trained immunity. *Cell Reports*.

[8] Kaufmann E., Sanz J., Dunn J. L. (2018). BCG educates hematopoietic stem cells to generate protective innate immunity against tuberculosis. *Cell*.

[9] Cirovic B., de Bree L. C. J., Groh L. (2019). Human Bacille Calmette-Guérin vaccination elicits trained immunity via the hematopoietic progenitor compartment. *SSRN Electronic Journal*.

[10] Ellouz F., Adam A., Ciorbaru R., Lederer E. (1974). Minimal structural requirements for adjuvant activity of bacterial peptidoglycan derivatives. *Biochemical and biophysical research communications.*.

[11] Huang Z., Wang J., Xu X. (2019). Antibody neutralization of microbiota-derived circulating peptidoglycan dampens inflammation and ameliorates autoimmunity. *Nature Microbiology*.

[12] Clarke T. B., Davis K. M., Lysenko E. S., Zhou A. Y., Yu Y., Weiser J. N. (2010). Recognition of peptidoglycan from the microbiota by Nod1 enhances systemic innate immunity. *Nature Medicine*.

[13] Girardin S. E., Boneca I. G., Viala J. (2003). Nod2 is a general sensor of peptidoglycan through muramyl dipeptide (MDP) detection. *The Journal of Biological Chemistry*.

[14] Ogawa C., Liu Y.-J., Kobayashi K. S. (2011). Muramyl dipeptide and its derivatives: peptide adjuvant in immunological disorders and cancer therapy. *Current Bioactive Compounds*.

[15] Divangahi M., Mostowy S., Coulombe F. (2008). NOD2-deficient mice have impaired resistance to Mycobacterium tuberculosis infection through defective innate and adaptive immunity. *Journal of Immunology*.

[16] Hansen J. M., Golchin S. A., Veyrier F. J. (2014). N-glycolylated peptidoglycan contributes to the immunogenicity but not pathogenicity of Mycobacterium tuberculosis. *The Journal of Infectious Diseases*.

[17] Assarsson E., Lundberg M., Holmquist G. (2014). Homogenous 96-plex PEA immunoassay exhibiting high sensitivity, specificity, and excellent scalability. *PLoS One*.

[18] Al Nabhani Z., Dietrich G., Hugot J. P., Barreau F. (2017). Nod2: the intestinal gate keeper. *PLoS Pathogens*.

[19] van der Meer J. H. M., Netea M. G., Dinarello C. A. (2009). Modulation of muramyl dipeptide stimulation of cytokine production by blood components. *Clinical and Experimental Immunology*.

[20] Kitaura H., Ishida M., Kimura K. (2018). Role of muramyl dipeptide in lipopolysaccharide-mediated biological activity and osteoclast activity. *Analytical Cellular Pathology*.

[21] Traub S., von Aulock S., Hartung T., Hermann C. (2016). Invited review: MDP and other muropeptides - direct and synergistic effects on the immune system. *Journal of endotoxin research.*.

[22] Hewitt R. E., Pele L. C., Tremelling M., Metz A., Parkes M., Powell J. J. (2012). Immuno-inhibitory PD-L1 can be induced by a peptidoglycan/NOD2 mediated pathway in primary monocytic cells and is deficient in Crohn’s patients with homozygous NOD2 mutations. *Clinical Immunology*.

[23] Liese A. M., Siddiqi M. Q., Siegel J. H., Denny T., Spolarics Z. (2001). Augmented TNF-alpha and IL-10 production by primed human monocytes following interaction with oxidatively modified autologous erythrocytes. *Journal of Leukocyte Biology*.

[24] Carrillo E. H., Gordon L. E., Richardson J. D., Polk H. C. (2002). Free hemoglobin enhances tumor necrosis Factor-*α* production in isolated human monocytes. *The Journal of Trauma: Injury, Infection, and Critical Care*.

[25] McFaul S. J., Bowman P. D., Villa V. M., Gutierrez-Ibanez M. J., Johnson M., Smith D. (1994). Hemoglobin stimulates mononuclear leukocytes to release interleukin-8 and tumor necrosis factor alpha. *Blood*.

[26] Johnson J. W., Fisher J. F., Mobashery S. (2013). Bacterial cell-wall recycling. *Annals of the New York Academy of Sciences*.

