# Supplementary figures and images for: BCG-Induced Trained Immunity in Healthy Individuals: The Effect of Plasma Muramyl Dipeptide Concentrations
Source: J Immunol Res. 2020 Jun 15;2020:5812743. doi: 10.1155/2020/5812743 (PMC7312554; doi:10.1155/2020/5812743)

Supplementary Figure 1

A.

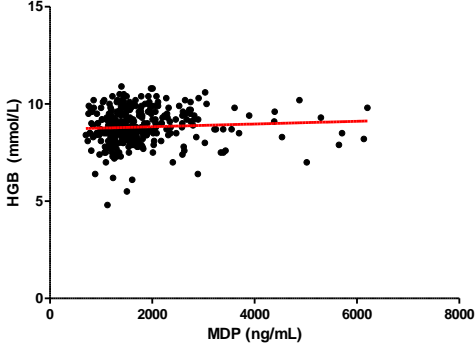

B.

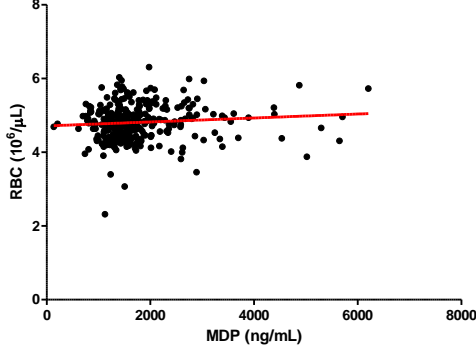

C.

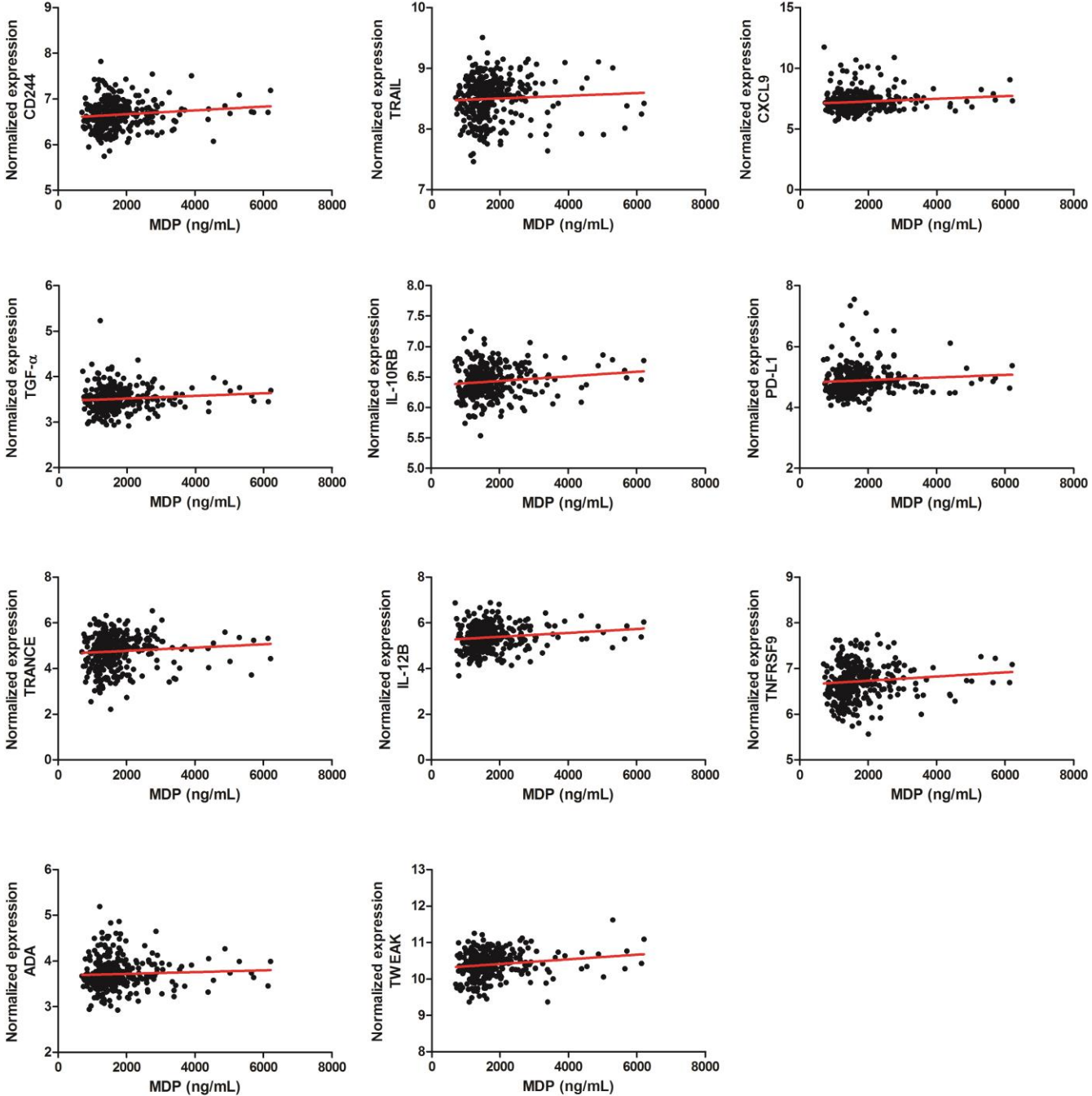

Supplementary Figure 2

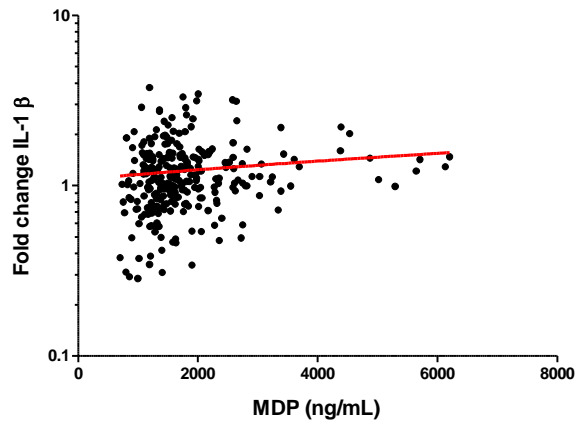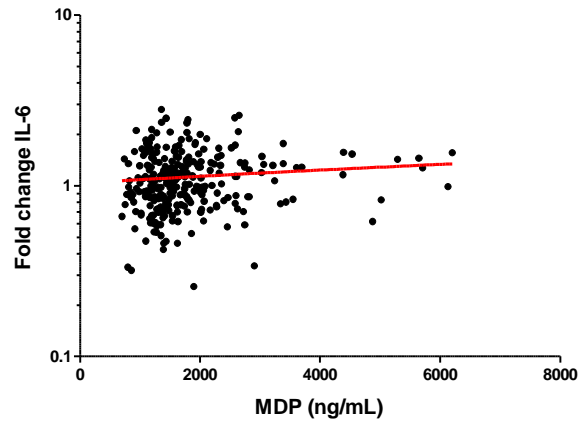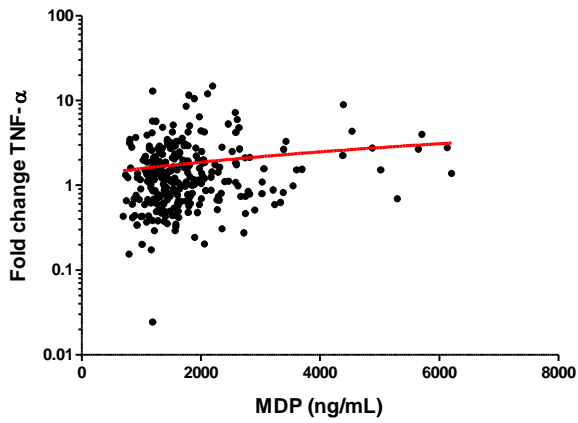

Supplementary Figure 3

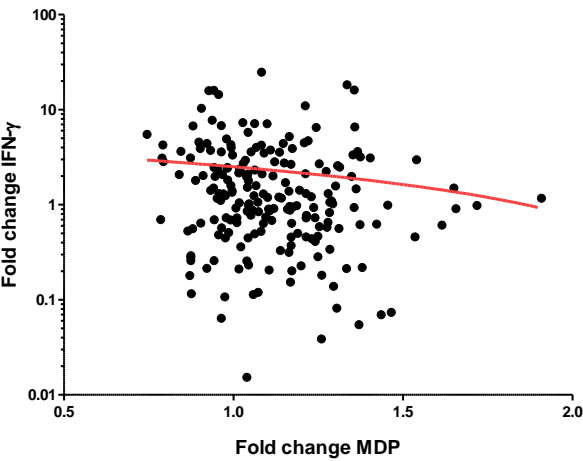

Supplement: Supplementary Materials — Supplementary Figure 1:Circulating MDP is positively correlated with the concentration of red blood cells and systemic inflammation. (A) Hemoglobin (HGB, mmol/L) and (B) amount of red blood cells (RBC, 10^6/μL) measured on Sysmex hematology analyzer correlated with MDP concentrations at baseline (n = 316). (C) Spearman correlation of circulating MDP concentrations and immunomodulators at baseline in peripheral blood by using Olink platform (n = 313). Supplementary Figure 2: Baseline MDP concentrations are associated with BCG-induced trained immunity responses. Spearman correlation of circulating MDP concentrations at baseline and fold changes of IL-1β, IL-6, and TNF-α production to S. aureus stimulation three months after BCG vaccination (n ≥ 289). Supplementary Figure 3: Changes in MDP concentration upon BCG vaccination correlate with changes in IFN-γ production upon ex vivo S. aureus stimulation. Circulating MDP concentrations before versus three months after BCG vaccination are correlated to IFN-γ production after 7 days upon ex vivo S. aureus stimulation (Spearman correlation, n = 212). [file 5812743.f1.pdf]
